# Data mining analysis of the prognostic impact of N^6^-methyladenosine regulators in patients with endometrial adenocarcinoma

**DOI:** 10.7150/jca.50868

**Published:** 2021-06-05

**Authors:** Junyu Zhai, Shang Li, Yu Li, Yanzhi Du

**Affiliations:** 1 Center for Reproductive Medicine, Ren Ji Hospital, School of Medicine, Shanghai Jiao Tong University, Shanghai 200135, China.; 2 Shanghai Key Laboratory for Assisted Reproduction and Reproductive Genetics, Shanghai 200135, China.; 3 Department of Gynecology and Obstetrics, Shanghai Jiao Tong University Affiliated Sixth People's Hospital, Shanghai 200233, China.

**Keywords:** N^6^-methyladenosine, Endometrial Adenocarcinoma, Energy Metabolism, RNA-Binding Protein

## Abstract

We reanalyzed the expression of 16 acknowledged N^6^-methyladenosine (m^6^A) RNA regulators in 406 endometrial adenocarcinoma patients and 19 controls using The Cancer Genome Atlas (TCGA) dataset, and further verified our results using Gene Expression Omnibus (GEO) dataset and real-time quantitative polymerase chain reaction. Thirteen m^6^A RNA methylation regulators were differentially expressed between patients with endometrial adenocarcinoma and controls. FTO, RBM15, and YTHDF1, were identified as independent prognostic markers and closely associated with International Federation of Gynecology and Obstetrics grade in endometrial cancer patients. GEO dataset also verified the differential expression of FTO and RBM15 between patients with endometrial adenocarcinoma and hyperplasia. Functional enrichment and ingenuity pathway analysis network suggested that FTO and RBM15 contributed to the survival of patients with endometrial adenocarcinoma via the regulation of connective tissue development, catabolic process, RNA stability, oxidative demethylation, temperature homeostasis, and energy metabolism through IGF1, IRS1, RBM24, LARP1, and CBFA2T3. The decreased *FTO* expression and increased *RBM15* expression in endometrial adenocarcinoma from our validation cohort was consistent with *in silico* analysis using TCGA and GEO datasets. In conclusion, m^6^A methylation regulators, especially FTO, RBM15, and YTHDF1, are critical in the progression and prognosis of endometrial adenocarcinoma.

## Introduction

As the most common gynecologic carcinoma 1, more than 80% of endometrial cancers are adenocarcinomas of endometrium, originating from endometrial cells that grow out of control. Endometrial carcinoma accounts for most cases of endometrial adenocarcinoma. In addition, serous adenocarcinoma, adenosquamous carcinoma, and uterine carcinosarcoma also belong to endometrial cancers 2. Although a considerable number of studies have investigated the potential mechanisms and biomarkers of endometrial cancers 3, 4, the etiology remains unclear. Novel methods for early detection and prognostic prediction for patients, especially those with advanced or recurrent endometrial cancers are essential to improve long-term outcomes of patients.

Aberrant gene expression, induced by epigenetic alterations, contributes to the development of endometrial cancers 5. Preliminary data suggests that DNA methylation biomarkers are useful in early diagnosis and risk prediction for high-risk populations of endometrial cancers 5, 6. DNA hypermethylation can be reversed by epigenetic inhibitors, leading to high expression of silenced genes and reversal of malignant biological behavior 7. Thus, epigenetics provides a potential target in the clinical prediction and management of endometrial cancer.

In addition to DNA methylation, the role of N^6^-methyladenosine (m^6^A) in cancers has recently attracted the attention of more and more researches. As the most abundant RNA modification in eukaryotic cells, m^6^A modifies approximately 0.1-0.4 % of all adenosines in RNA, accounting for about 50 % of total methylated ribonucleotides 8. m^6^A is a dynamic and reversible epigenetic modification, whose dynamic methylation and biological function are controlled by “writers” “erasers”, and “readers” that promote, remove, and exert the functions of m^6^A-modified sites, respectively 8. “Writers” are composed of several methyltransferase including methyltransferase-like 3 (METTL3), METTL14, METTL16, Wilms' tumor 1-associated protein (WTAP), RNA-binding motif protein 15 (RBM15), KIAA1429, and zinc finger CCCH-type containing 13 (ZC3H13), etc. 9, 10. “Erasers” are demethylases that catalyze m^6^A demethylation, among which fat mass and obesity-associated protein (FTO) is firstly identified 11, followed by α-ketoglutarate-dependent dioxygenase homolog 5 (ALKBH5) 12. Interaction with variable “readers” is required in the m^6^A modification to exert different downstream biological functions. YT521-B homology (YTH) domain family members are the main “readers” which regulate nuclear export, RNA splicing, decay, and translation 13, 14. Besides, certain heterogeneous nuclear ribonucleoprotein (HNRNP) family members and insulin-like growth factor 2 mRNA binding proteins (IGF2BPs) could also recognize the m^6^A modification site and mediate the functions of m^6^A 15, 16.

Accumulating evidence has revealed the crucial role of m^6^A modification in numerous physiological processes, especially tumor initiation and progression, of glioblastoma, acute myeloid leukemia, hepatocellular and breast carcinoma 17, 18. Recently, decreased m^6^A RNA methylation level caused by METTL14 R298 mutation or decreased METTL3 expression has been reported to promote tumorigenicity and cell proliferation via activation of AKT signaling pathway in endometrial cancer 18. This research identifies the function of m^6^A RNA methylation in the pathogenesis of endometrial cancer. However, a comprehensive and systematic analysis of the expression of m^6^A RNA methylation regulators in endometrial cancer as well as their prognostic value is currently lacking.

Herein, we reanalyzed the expression of 16 acknowledged m^6^A RNA regulators in 406 endometrial adenocarcinoma patients and 19 controls using The Cancer Genome Atlas (TCGA) dataset to stratify the prognosis of endometrial adenocarcinoma. Subsequently, microarray data from the publicly available Gene Expression Omnibus (GEO) dataset and real-time quantitative polymerase chain reaction (RT-qPCR) using clinical samples were utilized to verify the differential expression of key m^6^A methylation regulators in patients with endometrial adenocarcinoma.

## Materials and Methods

### Datasets

The expression data and corresponding clinical characteristics for 406 patients with endometrial adenocarcinoma and 19 controls were downloaded from TCGA dataset (http://cancergenome.nih.gov/). We screened suitable clinical samples from the TCGA database with the terms “corpus uteri”, “adenocarcinoma”, and “TCGA” as the inclusion criteria; thereby 406 patients and 19 controls from studies meeting the database requirements were included in our further analysis. Controls were from tumor adjacent normal endometrium or women without endometrial cancer. The only common information for these enrolled patients were age, International Federation of Gynecology and Obstetrics (FIGO) grade, and survival information, so all patients' ages and tumor FIGO grades were sorted and listed in Table S1.

For further validation, the expression data from 64 endometrial adenocarcinoma and 33 endometrial hyperplasia tissues was downloaded from GSE 106191 of the GEO database (https://www.ncbi.nlm.nih.gov/geo/). All 33 endometrial hyperplasia tissues were adjacent to endometrial adenocarcinoma. The clinical parameters of these endometrial adenocarcinoma and hyperplasia patients have been presented previously 19. Gene expression dataset was downloaded from the Affymetrix Human Genome U133 Plus 2.0 Array (HG-U133_Plus_2).

### Analysis of m^6^A RNA methylation regulators

According to published literature 20, we assembled 18 m^6^A RNA methylation regulators. We further selected 16 regulators among them due to available data from GSE 106191 and TCGA database. We analyzed the expression of these 16 genes between women with and without endometrial adenocarcinoma from TCGA and GEO datasets independently using the Wilcoxon test in R software. Correlations between m^6^A methylation regulators were identified using Spearman correlation in the “Corrplot” package of R software. P < 0.001 was considered as significantly correlated to each other.

### Bioinformatic analysis of TCGA dataset

Univariate Cox regression analyses of the expression of m^6^A RNA methylation regulators in endometrial adenocarcinoma using TCGA dataset to determine their prognostic value. Four genes were identified related with patients' survival (P < 0.1), and were further selected for functional analysis and potential risk signature using least absolute shrinkage and selection operator (LASSO) Cox regression algorithm. Eventually, three genes and their coefficients were identified, selecting the best penalty parameter λ associated with the smallest 10-fold cross validation within TCGA database 21. A risk score was calculated for the prognosis of each patient in TCGA dataset using the formula: risk score = ΣCoefi∗xi. Risk scores in patients with different clinical features were compared using one-way analysis of variance or t-test. We applied multivariate Cox regression analysis to verify prognostic value of risk score and clinical features.

According to risk score (higher or lower than the median value), patients with endometrial adenocarcinoma were classified into two groups, namely high-risk and low-risk, respectively. The survival of patients in two groups was compared using Kaplan-Meier method with a two-sided log-rank test. The functional enrichment of differentially expressed genes (DEGs) between two groups was determined using Gene Ontology (GO) enrichment and Kyoto Encyclopedia of Genes and Genomes (KEGG) enrichment.

### Identification of DEGs of GEO dataset

After downloading GSE 106191 from GEO database, the “impute” R software package was utilized to impute missing expression data, while the “limma” R software package was utilized to normalize gene expression and to identify the DEGs between endometrial adenocarcinoma and endometrial hyperplasia groups. P < 0.01 denoted significant difference.

### Weighted gene co-expression network analysis (WGCNA) and functional enrichment from GEO dataset

We did WGCNA to analyze the DEGs in GEO dataset and identified the relationships among *FTO*, *RBM15*, and their potential target genes in women with endometrial adenocarcinoma. GO analysis was utilized to visualize the potential molecular function and biological significance of DEGs in the same module with *FTO* and *RBM15*, whereas KEGG pathway enrichment was used to analyze the potential functions of these genes. The gene ID was set using the “org.Hs.eg.db” of R software and, subsequently, GO and KEGG analyses were carried out with “clusterProfiler”.

### Ingenuity pathway analysis (IPA)

IPA was performed to identify the possible target genes of *FTO* and network, which might participate in the progression of endometrial adenocarcinoma.

### Clinical sample collection

In order to validate the *in silico* analysis results, we collected 30 clinical samples of untreated endometrial adenocarcinoma and controls (N=15 for each group) in Department of Gynecology and Obstetrics, Shanghai Jiao Tong University Affiliated Sixth People's Hospital, Shanghai Jiao Tong University School of Medicine. The normal endometrial tissues as controls were collected from hysteroscopy biopsy in patients of fibroids, polyps, or infertility. The inclusion criteria of enrolled endometrial adenocarcinoma were as follows: patients were diagnosed with stages I-II endometrial cancer according to the staging system of FIGO 2009 guidelines and the histotype is adenocarcinoma. Patients with other malignancies and those who had received chemotherapy or radiotherapy before surgery were excluded. Informed consent was obtained from all participants. All procedures were reviewed and approved by the ethics committees of Institutional Review Board in Shanghai Sixth People's Hospital affiliated for Shanghai Jiao Tong University. Fresh endometrial tumors and normal endometrial tissues were separately dissected at the time of surgery and immediately transferred to liquid nitrogen for storage. The clinical parameters for recruited patients are presented in Table S2.

### Real-time quantitative polymerase chain reaction (RT-qPCR)

Total RNA from endometrial tissues was extracted using a Total RNA Isolation Kit (FOREGENE, Chengdu, China) and then reversely transcribed into cDNA (TAKARA, Dalian, China). The mRNA expression of *METTL3*,* FTO*, *YTHDF1*, and *RBM15* was detected using RT-qPCR. Results were analyzed by ΔΔCt method. The ratio of the target gene over *β-ACTIN* was calculated as the target mRNA level. The primer sequences used for targeting genes were as follows:

*METTL3* (human), 5'-TTGTCTCCAACCTTCCGTAGT-3' (forward) and 5'-CCAGATCAGAGAGGTGGTGTAG-3' (reverse).

*FTO* (human), 5'-ACTTGGCTCCCTTATCTGACC-3' (forward) and 5'-TGTGCAGTGTGAGAAAGGCTT-3' (reverse).

*YTHDF1* (human), 5'-GTGCTGATAGATGTTGTTCCCC-3' (forward) and 5'-ATACCTCACCACCTACGGACA-3' (reverse).

*RBM15* (human), 5'-GTGAGGACTCGACTTCCCG-3' (forward) and 5'-GCCGCTATCGGTCTTTCCG-3' (reverse).

*β-ACTIN* (human), 5'-GGGAAATCGTGCGTGACATTAAG-3' (forward) and 5'-TGTGTTGGCGTACAGGTCTTTG -3' (reverse).

### Statistical analysis

Results are expressed as mean ± SEM or SD. We used paired Student's t-test or one-way analysis of variance followed by the Newman-Keuls multiple comparison tests. Statistical significance is shown as *P < 0.05, **P < 0.01, and ***P < 0.001.

## Results

### Altered expression of m^6^A RNA methylation regulators in endometrial adenocarcinoma

**Figure 1:**
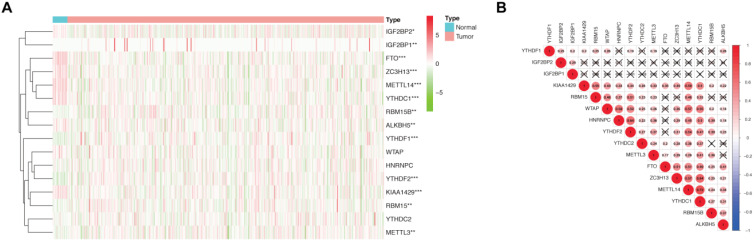
** Expression and correlation of 16 m^6^A methylation regulators in patients with endometrial adenocarcinoma and controls. (A)** Expression of 16 m^6^A methylation regulators in patients with endometrial adenocarcinoma (N=406) and controls (N=19) from TCGA dataset. * P < 0.05, ** P < 0.01, *** P < 0.001.** (B)** Spearman correlation analysis of 16 m^6^A methylation regulators. P < 0.001 denotes statistical significance.

For the first time, we explored the expression of 16 established m^6^A methylation regulators, including writers, erasers, and readers, in endometrial adenocarcinoma using TCGA dataset. For writers, the expression of *METTL3* and *RBM15/15B* was significantly increased in patients with endometrial adenocarcinoma versus controls. In contrast, the expression of *METTL14*, *KIAA1429*, and *ZC3H13* was drastically decreased in patients with endometrial adenocarcinoma (Figure 1A). Furthermore, aberrant expression of erasers was also detected, and both *FTO* and *ALKBH5* were significantly decreased in women with endometrial adenocarcinoma versus controls (Figure 1A). Therefore, altered total m^6^A level induced by aberrant methylase and demethylase might contribute to the development of endometrial adenocarcinoma. Additionally, the expression of readers, biological function mediators of m^6^A, was also changed in patients with endometrial adenocarcinoma. The expression of *YTHDF1*, *YTHDF2*, and *IGF2BP1/2* was increased in women with endometrial adenocarcinoma, whereas a reduction in *YTHDC1* was detected (Figure 1A). Since RNA splicing, translation, and decay could be regulated by the combination of dysregulated readers, aberrant m^6^A methylation site might give rise to dysregulation of target genes in endometrial adenocarcinoma.

Spearman correlation was subsequently used to clarify the possible co-expression and association between 16 regulators. *KIAA1429* was closely related to 13 m^6^A methylation regulators, while *METTL14* and *YTHDC1* were associated with 12 regulators (Figure 1B). Combined with the differential expression and correlation results, we hypothesized that these aberrant m^6^A methylation regulators might contribute to the pathogenesis of endometrial adenocarcinoma.

### Prognostic value and risk signature of m^6^A methylation regulators in endometrial adenocarcinoma

**Figure 2:**
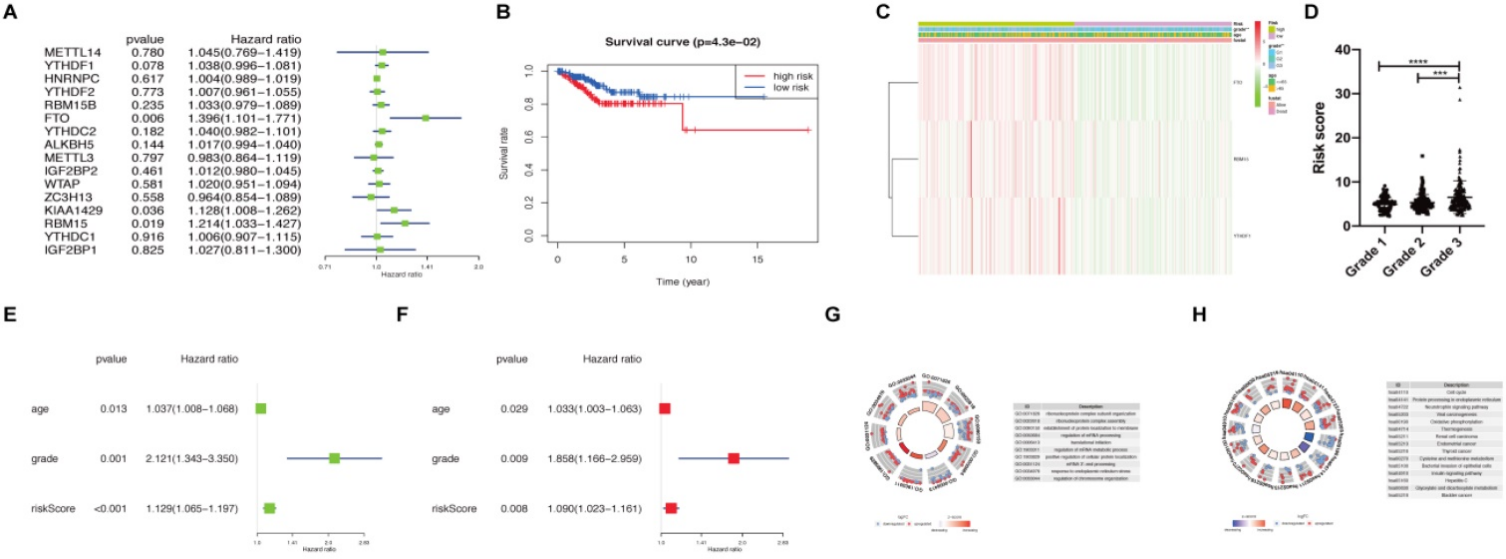
** Risk signature constructed using m^6^A methylation regulators. (A)** Process of constructing risk signature using 3 m^6^A methylation regulators. Hazard ratio, 95% confidence intervals analyzed by univariate Cox regression. **(B)** Kaplan-Meier overall survival curves in patients with endometrial adenocarcinoma (N=406) of TCGA dataset between high-risk and low-risk groups according to risk score using our predictive signature after LASSO regression. **(C)** Heatmap of the expression of *FTO*, *RBM15*, and *YTHDF1* between high-risk and low-risk groups. The distribution of clinical features was also presented. **(D)** The risk score of patients with different FIGO grades. **(E-F)** Univariate (E) and multivariate (F) Cox regression analyses of correlations between age, grade, risk score, and survival of endometrial adenocarcinoma patients. **(G-H)** GO and KEGG enrichment of DEGs in patients with endometrial adenocarcinoma between high-risk and low-risk groups. ** P < 0.01, *** P < 0.001, **** P < 0.0001.

We applied univariate Cox regression analysis to investigate the prognostic value of 16 m^6^A methylation regulators using their expression in patients with endometrial adenocarcinoma in TCGA dataset (N=406) (Figure 2A). Three of the 16 tested genes (*FTO*, *KIAA1429*, and *RBM15*) were evidently correlated with overall survival (P < 0.05) and had a hazard ratio > 1. We further performed LASSO Cox regression algorithm to predict the clinical outcomes of endometrial adenocarcinoma patients via *FTO*, *KIAA1429*, and *RBM15*. Since the number of genes was insufficient for regression, *YTHDF1* (P < 0.1) was also added into the prediction model. That is to say, a total of four genes (P < 0.1 in univariate Cox regression) were applied to LASSO regression. We picked 3 genes to construct the risk signature, and the coefficients of *FTO*, *RBM15*, and *YTHDF1* were 0.24, 0.13, and 0.02, respectively. The survival curve of high- and low- expression groups for each gene was presented in Figure S1.

Subsequently, we calculated the risk score for patients with endometrial adenocarcinoma using coefficients of these 3 predicted genes. All patients with endometrial adenocarcinoma were divided into two groups based on risk score, namely as high-risk and low-risk. A distinct difference in overall survival between the two groups was identified in Figure 2B (P < 0.05). We further analyzed the expression of *FTO*, *RBM15*, and *YTHDF1* in two groups using TCGA dataset (Figure 2C). An apparent difference was observed between two groups with respect to the International Federation of Gynecology and Obstetrics (FIGO) grade (P < 0.01) (Figure 2C). We further analyzed the association between risk scores and age, as well as FIGO grade; the risk score was significantly increased in grade 3 compared with those in grade 1 and grade 2 (P < 0.001) (Figure 2D).

Moreover, univariate and multivariate Cox regression analyses were applied to verify whether the risk signature could be independently identified as the prognostic indicator. Age, FIGO grade, and risk score remained closely correlated with survival no matter by univariate Cox regression analysis (Figure 2E) or multivariate analysis (Figure 2F). Finally, we analyzed DEGs between high-risk and low-risk groups and utilized functional enrichment of GO and KEGG to clarify the potential target functions and pathway of m^6^A methylation regulators. The biological process enriched from DEGs focused on RNA processing, translation initiation, and protein localization, which are main functions of m^6^A (Figure 2G). KEGG analysis enriched the altered expression of genes in the cell cycle, oxidative phosphorylation, thermogenesis, carcinogenesis, and protein processing (Figure 2H). Hence, *FTO*, *RBM15*, and *YTHDF1* may regulate the RNA processing and translation of target genes involved in the cell cycle, thermogenesis and other pathways, leading to the tumor process and prognosis of endometrial adenocarcinoma.

### Validation of m^6^A methylation regulators and potential target networks

**Figure 3:**
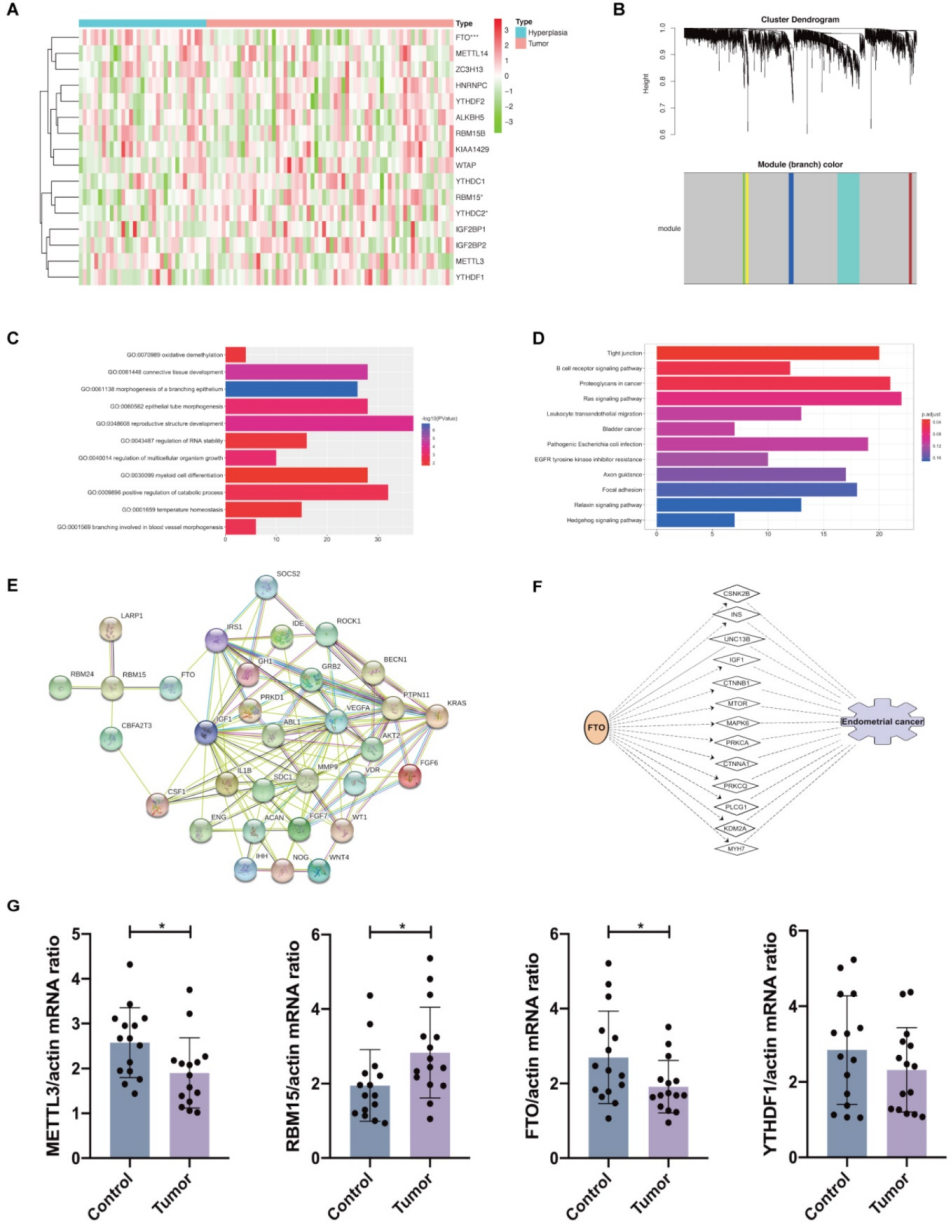
** Expression of 16 m^6^A methylation regulators in patients with and without endometrial adenocarcinoma. (A)** Expression of 16 m^6^A methylation regulators in patients with endometrial adenocarcinoma (N=64) and hyperplasia (N=33) from GEO dataset. **(B)** WGCNA analysis of DEGs between women with and without endometrial adenocarcinoma. **(C-D)** GO and KEGG enrichment of DEGs belonging to the same module as *RBM15* and *FTO*. GO composed of 11 biological processes in which *FTO* and *RBM15* were involved. **(E)** The protein-protein interaction network of FTO, RBM15, and other 30 target protein involved in the above 11 biological processes. **(F)** Identification of the possible target genes of *FTO* and network that may participate in the progression of endometrial adenocarcinoma via IPA. * P < 0.05, *** P < 0.001. **(G)** The mRNA expression of *METTL3*, *RBM15*, *FTO*, and *YTHDF1* in endometrial adenocarcinoma and control endometrium samples (N=15 for each group). * P < 0.05.

The gene expression profiles of 33 hyperplasia tissues and 64 endometrial adenocarcinoma tissues were obtained from GSE 106191. *FTO* expression was significantly decreased, whereas *RBM15* was increased (Figure 3A), similar to results in TCGA dataset. From TCGA and GEO datasets, we found that *FTO* and *RBM15* might take critical roles in the prognosis of endometrial adenocarcinoma. The co-expression of DEGs between women with and without endometrial adenocarcinoma was further detected using WGCNA. We found that both *RBM15* and *FTO* belonged to the gray module (threshold = 0.9; Figure 3B). All 1315 DEGs in the gray module were enriched via GO and KEGG analyses, and we identified 11 biological processes in which *FTO* and *RBM15* were involved. Thus, *FTO* and *RBM15* were suggested to contribute to the progression of endometrial adenocarcinoma through these 11 biological processes, including connective tissue development, reproductive structure development, positive regulation of catabolic process, regulation of multicellular organism growth, regulation of RNA stability, oxidative demethylation, and temperature homeostasis, etc. (Figure 3C and 3D).

Furthermore, the interactions among 127 DEGs involved in these 11 biological processes were analyzed using STRING. We observed that *RBM15* interacted with *la ribonucleoprotein 1* (*LARP1*), *RBM24*, *CBFA2/RUNX1 partner transcriptional co-repressor 3* (*CBFA2T3*), and *FTO*; meanwhile, *FTO* interacted with *insulin-like growth factor 1* (*IGF1*), *insulin receptor substrate 1* (*IRS1*), and *RBM15* (Figure 3E). Therefore, we supposed that *FTO* facilitated the progression of endometrial adenocarcinoma via the functions of *IGF1* and *IRS1*. The potential target genes mediating the regulation of *RBM15* and *FTO* to the progression of endometrial adenocarcinoma have been presented in Table 1. Then, we used IPA to predict the possible target genes and network through which *FTO* led to the progression of endometrial adenocarcinoma. *IGF1* was also shown to mediate the functional role of *FTO* in endometrial adenocarcinoma (Figure 3F). Hence, our results provided a potential mechanism and network for the regulation of *FTO* and *RBM15* to the progression of endometrial adenocarcinoma.

Finally, we validated the mRNA expression of *METTL3*, *FTO*, *RBM15*, and *YTHDF1* in clinical samples of endometrial tissues from endometrial adenocarcinoma patients and controls. We found that *METTL3* and *FTO* mRNA expression was evidently decreased in endometrial adenocarcinoma while *RBM15* mRNA expression was increased with no changes in *YTHDF1* expression in cases versus controls (Figure 3G). These results were consistent with previous *in silico* analysis using TCGA and GEO datasets.

## Discussion

As the fourth most common malignancy in females, endometrial cancer presents a high rate of recurrence and poor prognosis in patients with advanced disease 5. Thus, early diagnosis and the identification of biomarkers associated with the prediction of prognosis are of vital importance in clinical management. Mainly focusing on DNA methylation or histone acetylation, epigenetics takes various roles in progression and diagnosis of endometrial cancer, as well as risk prediction and identification of treatment targets 22, 23. In the present research, we elucidated that m^6^A RNA methylation regulators, especially *FTO*, *RBM15*, and *YTHDF1*, were likewise closely related with prognosis of endometrial adenocarcinoma. Tumor grade is the description of a tumor based on the differentiation of its cells. Grade 1 indicates a well-differentiated tumor, while grade 3 is often associated with rapid grow and migration, as well as poor prognosis 24. According to our risk signature, risk score was positively correlated with tumor grade, further demonstrating the prognostic value of our m^6^A regulator model in endometrial adenocarcinoma. Moreover, we also validated the roles of FTO and RBM15 in the prognosis of endometrial adenocarcinoma using the GEO dataset and predicted the possible target genes and biological processes of FTO and RBM15, which might contribute to the regulation of these two genes to endometrial adenocarcinoma. Thus, we provided a prediction biomarker and a risk signature that have the potential to predict the prognosis of patients with endometrial adenocarcinoma; this may be beneficial to clinical diagnosis and management.

Reductions in m^6^A methylation induced by decreased *METTL3* and mutation in *METTL14* activate the phosphoinositide 3 kinase/protein kinase B (PI3K/AKT) pathway, promoting proliferation and tumorigenicity of endometrial cancer 18. In our study, the expression of *METTL14* and *KIAA1429*, methylase enzymes promoting methylation of the m^6^A site, was significantly decreased in endometrial adenocarcinoma. This result was consistent with the reduction of m^6^A levels in endometrial cancer observed in a previous study 18. However, decreased* FTO* expression in the tumor shown in our study was in contrast to its results. We supposed that the type of endometrial cancer used in the research might account for this difference. We selected endometrial adenocarcinoma, while the previous study reported by Liu *et al* investigated a mix of endometrioid adenocarcinoma, endometrioid, clear-cell carcinoma, and endometrioid with squamous differentiation. Additionally, difference noted in tissues might also be responsible for the discrepancy. The previous study compared the tumor with the adjacent tissue, whereas we chose tissues from patients with and without tumor.

Endometrial cancer is an estrogen-dependent disease; both hormonal dysregulation and obesity can facilitate the development and progression of this disease. Obesity (BMI >30 kg/m^2^) is considered as the greatest risk factor for patients with endometrial cancer 25. Weight loss intervention involving metabolic surgery is an alternative to standard surgical treatment 25, 26. Herein, we built a risk prediction model using FTO, m^6^A methylation regulator closely associated with body mass, obesity, and energy metabolism 27. FTO is reported to suppress mitochondrial thermogenesis in the adipocyte precursor cells 28. According to our prediction model, pathways of thermogenesis were also enriched in DEGs between high-risk and low-risk patients with endometrial adenocarcinoma (Figure 2H). Furthermore, FTO proves to promote glycolysis though PI3K/AKT pathway in breast cancer cells 29 and PI3K/AKT signaling pathway was also enriched in our DEGs. In our results, IGF1 and IRS1, essential factors in the glucose metabolism, thermogenesis, and glucose catabolic process 30, were considered as the potential target genes of FTO in the regulation of endometrial adenocarcinoma (Figure 3E and 3F). Hence, we supposed that the altered expression and variants of the *FTO* gene contribute to the progression and prognosis of women with endometrial cancer through energy metabolism as well as IGF1 signaling. However, the underlying mechanism warrants further investigation.

Besides of energy disorder, FTO participates in the regulation of prognosis of cancer through several other ways. A previous study demonstrates that reduction of *FTO* upregulates *cyclin D1* RNA methylation, which in turn accelerates *cyclin D1* mRNA degradation and results in inhibition of G1 progression and cell cycle 31. Interestingly, according to the results of our analysis, DEGs between high- and low-risk patients with endometrial cancer were also enriched in cell cycles (Figure 2H). In addition, IPA network also indicated the regulation of lysine demethylase 2A (KDM2A) by FTO (Figure 3F), which may arrest cells in the G2/M phase. Thus, FTO may regulate cell cycle-associated genes in the progression of endometrial cancer. In addition, decreased FTO takes a tumorigenic role in bladder cancer 32 and a highly selective inhibitor of FTO could restrain the cisplatin-induced cytotoxicity in bladder cancer cells 33. Epithelial-to-mesenchymal transition (EMT) is a vital process that drives, tumor initiation and metastasis, resistance to anoikis, and refractory response to chemotherapy, contributing to the prognosis of patients with cancer 34. IGF1 signaling contributes to the regulation of EMT 35, and our result also indicated the potential function of IGF1 in the regulatory role of FTO in endometrial cancer. Hence, FTO may be involved in the survival and prognosis of patients by regulating the sensitivity to cisplatin-based chemotherapy and the EMT. The decreased expression of *FTO* and co-expression of *IGF1* and *FTO* were also validated in the GEO dataset, further demonstrating the reliability of our analysis.

Apart from FTO, RBM15, a writer promoting the methylation of m^6^A site, also contributed to our risk signature. Our study suggested that RBM15 might give rise to epithelial tube morphogenesis, branching involved in blood vessel morphogenesis, myeloid cell differentiation, and morphogenesis of a branching epithelium. Consistent with our results, increased RBM15 is also associated with prognosis of lung adenocarcinoma 36. Additionally, YTHDF1 is a reader that can modulate the translation dynamics of m^6^A-modified mRNA 37 with prognostic value in lung adenocarcinoma 38. Spearman correlation analysis did not reveal any significant association among the expression of *FTO*, *RBM15*, and *YTHDF1*.

In summary, analysis of TCGA patient dataset, correlation analysis, and construction of a risk signature using m^6^A methylation regulators revealed their importance in the progression and prognosis prediction of endometrial adenocarcinoma, especially highlighting the roles of *FTO*, *RBM15*, and *YTHDF1*. Functional enrichment and IPA network suggested that FTO and RBM15 contributed to the survival of patients with endometrial cancer via *IGF1*, *IRS1*, *RBM24*, *LARP1*, and *CBFA2T3* under the regulation of connective tissue development, catabolic process, RNA stability, oxidative demethylation, temperature homeostasis, and energy metabolism, etc. Combinational therapy that targets FTO to promote the therapeutic effect and prolong survival may represent a personalized attractive treatment approach for patients with endometrial cancer. Further *in vivo* and *in vitro* studies are needed to validate our hypothesis.

## Supplementary Material

Supplementary figures and tables.

## Figures and Tables

**Table 1:** Potential target genes that mediate the regulation of RBM15 and FTO to the progression of endometrial cancer

Genes	Biological Process
RBM24	Positive regulation of catabolic process
CBFA2T3	Myeloid cell differentiation
LARP1	Positive regulation of catabolic process
IGF1	Positive regulation of catabolic process/regulation of multicellular organism growth
VEGFA	Reproductive structure development/temperature homeostasis/epithelial tube morphogenesis/branching involved in blood vessel morphogenesis/myeloid cell differentiation
IRS1	Positive regulation of catabolic process
GRB2	Reproductive structure development
PTPN11	Reproductive structure development
PRKD1	Positive regulation of catabolic process
GH1	Regulation of multicellular organism growth
ACAN	Connective tissue development
NOG	Connective tissue development/ morphogenesis of a branching epithelium
AKT2	Positive regulation of catabolic process
WT1	Connective tissue development/ reproductive structure development
ABL1	Epithelial tube morphogenesis
SDC1	Reproductive structure development
MMP9	Myeloid cell differentiation
SOCS2	Regulation of multicellular organism growth
CSF1	Connective tissue development/ epithelial tube morphogenesis
ENG	Epithelial tube morphogenesis
VDR	Reproductive structure development
ROCK1	Positive regulation of catabolic process
BECN1	Positive regulation of catabolic process
FGF6	Connective tissue development
FGF7	Morphogenesis of a branching epithelium
IDE	Positive regulation of catabolic process
IHH	Connective tissue development/epithelial tube morphogenesis
IL1B	Temperature homeostasis/positive regulation of catabolic process
KRAS	Epithelial tube morphogenesis/morphogenesis of a branching epithelium
WNT4	Morphogenesis of a branching epithelium/epithelial tube morphogenesis/reproductive structure development

All the biological processes were annotated as GO term enrichment analysis from DAVID Bioinformatics Resources 6.8 (https://david.ncifcrf.gov/).

## Abbreviations

m^6^A: N^6^-methyladenosine
METTL3: methyltransferase-like 3
METTL14: methyltransferase-like 14
METTL16: methyltransferase-like 16
WTAP: Wilms' tumor 1-associated protein
RBM15: RNA-binding motif protein 15
ZC3H13: zinc finger CCCH-type containing 13
FTO: fat mass and obesity-associated protein
ALKBH5: α-ketoglutarate-dependent dioxygenase homolog 5
YTH: YT521-B homology
HNRNP: heterogeneous nuclear ribonucleoprotein
IGF2BPs: insulin-like growth factor 2 mRNA binding proteins
TCGA: The Cancer Genome Atlas
GEO: Gene Expression Omnibus
RT-qPCR: real-time quantitative polymerase chain reaction
LASSO: least absolute shrinkage and selection operator
DEGs: differentially expressed genes
GO: Gene Ontology
KEGG: Kyoto Encyclopedia of Genes and Genomes
WGCNA: weighted gene co-expression network analysis
IPA: ingenuity pathway analysis
FIGO: International Federation of Gynecology and Obstetrics
LARP1: la ribonucleoprotein 1
CBFA2T3: CBFA2/RUNX1 partner transcriptional co-repressor 3
IGF1: insulin-like growth factor 1
IRS1: insulin receptor substrate 1
PI3K/AKT: phosphoinositide 3 kinase/protein kinase B
KDM2A: lysine demethylase 2A
EMT: epithelial-to-mesenchymal transition
