# Supplementary material for: Data mining analysis of the prognostic impact of N6-methyladenosine regulators in patients with endometrial adenocarcinoma
Source: J Cancer. 2021 Jun 5;12(15):4729–38. doi: 10.7150/jca.50868 (PMC8210566; doi:10.7150/jca.50868)
Supplement: Supplementary file 1 — Supplementary figures and tables. [file jcav12p4729s1.pdf]

Supplementary Material for

Data mining analysis of the prognostic impact of N^6-methyladenosine regulators in patients with endometrial adenocarcinoma

Figure S1

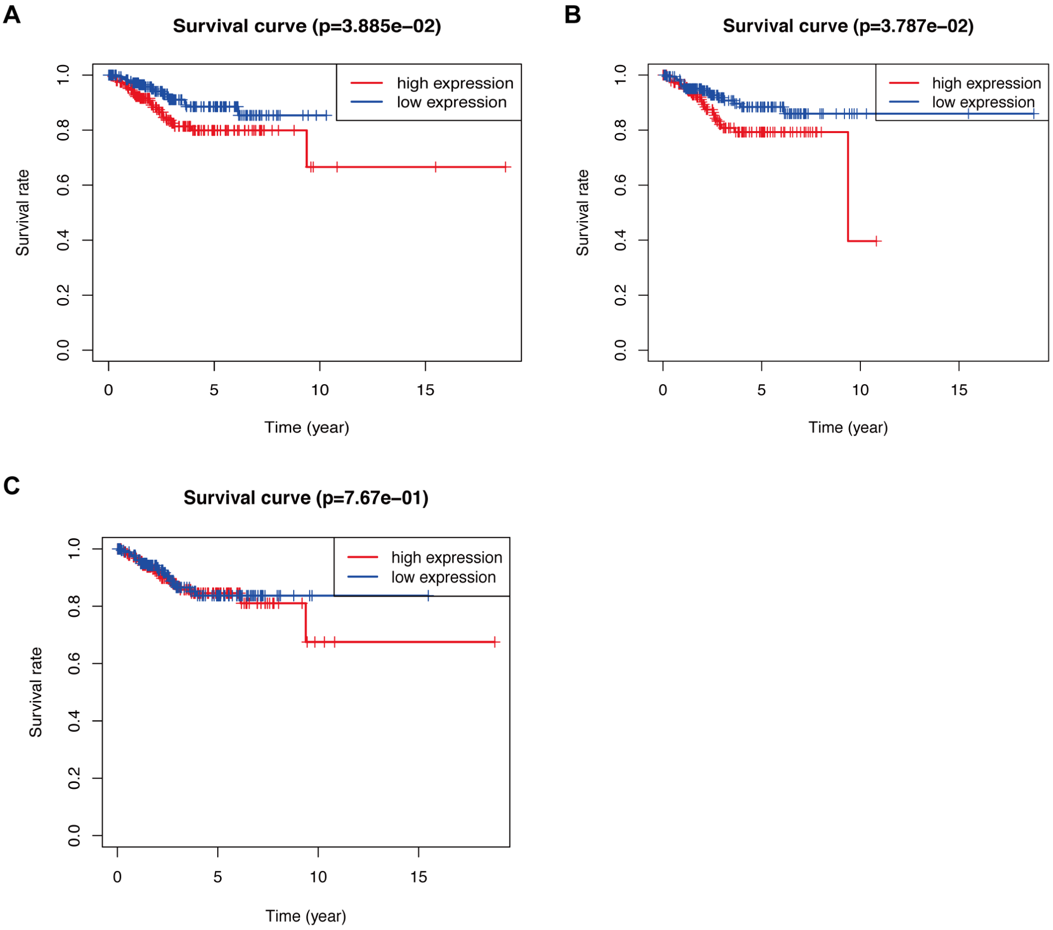

Figure S1. Kaplan–Meier overall survival curves for patients in TCGA datasets (only tumor, N=406) classified based on high and low expression of *FTO* (A), *RBM15* (B), and *YTHDF1* (C), respectively.

Table S1. Clinical parameters of patients with endometrial adenocarcinoma (TCGA dataset)

		Number (n=406)	Percentage (%)
**Age (31-90)**	＜Median	215	52.96
	≥Median	191	47.04
**Grade**	G1	97	23.89
	G2	119	29.31
	G3	190	46.80

Table S2. Clinical parameters of recruited patients with endometrial adenocarcinoma and controls (validation cohort)

	**Patients with endometrial adenocarcinoma**	**Patients with normal endometrial tissues**
**Number**	15	15
**Age**	47.60±6.82	43.87±6.94
**Menopause**		
Yes	10	4
No	5	11
**Pregnancy history**		
Yes	13	11
No	2	4
**Family history**		
Yes	1	—
No	14	—
**Clinical stage (FIGO)**		
I	12	—
II	3	—
III	0	
**Vascular invasion**		
Yes	0	—
No	15	—
**Lymph node metastasis**		
Yes	0	—
No	15	—

All data are Mean ± SD values.

FIGO, International Federation of Gynecology and Obstetrics.
